# Supplementary material for: Crawling and Gliding: A Computational Model for Shape-Driven Cell Migration
Source: PLoS Comput Biol. 2015 Oct 21;11(10):e1004280. doi: 10.1371/journal.pcbi.1004280 (PMC4619082; doi:10.1371/journal.pcbi.1004280)
Supplement: S1 Code — (ZIP) [file pcbi.1004280.s012.zip › release/tst/doc/html/functions_s.html]

Tissue Simulation Toolkit: Class Members


Tissue Simulation Toolkit
 0.1.4.1


- Main Page
- Namespaces
- Classes
- Files

- Class List
- Class Hierarchy
- Class Members

- All
- Functions
- Variables
- Related Functions

- a
- b
- c
- d
- e
- f
- g
- i
- j
- l
- m
- n
- o
- p
- q
- r
- s
- t
- v
- w
- x
- y
- z
- ~

Here is a list of all class members with links to the classes they belong to:

### - s -

- saturation
  : Parameter
- SearchNandPlot()
  : CellularPotts
- SearchNeighbours()
  : CellularPotts
- secr\_rate
  : Parameter
- Secrete()
  : PDE
- SetCellOwner()
  : Dish
- SetColour()
  : Cell
- SetGrad()
  : Cell
- SetJ()
  : Cell
- SetRandomTypes()
  : CellularPotts
- SetTargetArea()
  : Cell
- SetTargetLength()
  : Cell
- setTau()
  : Cell
- setValue()
  : PDE
- ShowDirections()
  : CellularPotts
- Sigma()
  : Cell
- sigma
  : Cell
- Sigma()
  : CellularPotts
- sigma
  : CellularPotts
- Sigma()
  : PDE
- sigma
  : PDE
- SimulationDone()
  : QtGraphics
- size\_init\_cells
  : Parameter
- SizeX()
  : CellularPotts
- sizex
  : CellularPotts
- SizeX()
  : Dish
- sizex
  : Parameter
- SizeX()
  : PDE
- sizex
  : PDE
- sizey
  : CellularPotts
- SizeY()
  : CellularPotts
  , Dish
- sizey
  : Parameter
  , PDE
- SizeY()
  : PDE
- spins\_converted
  : CellularPotts
- storage\_stride
  : Parameter
- store
  : Parameter
- subfield
  : Parameter
- sum\_x
  : Cell
- sum\_xx
  : Cell
- sum\_xy
  : Cell
- sum\_y
  : Cell
- sum\_yy
  : Cell


---

Generated on Thu Aug 14 2014 22:04:02 for Tissue Simulation Toolkit by  

 1.8.6
